# Greater retention in care among adolescents on antiretroviral treatment accessing “Teen Club” an adolescent‐centred differentiated care model compared with standard of care: a nested case–control study at a tertiary referral hospital in Malawi

**DOI:** 10.1002/jia2.25028

**Published:** 2017-11-27

**Authors:** Rachel K MacKenzie, Monique van Lettow, Chrissie Gondwe, James Nyirongo, Victor Singano, Victor Banda, Edith Thaulo, Teferi Beyene, Mansi Agarwal, Allyson McKenney, Susan Hrapcak, Daniela Garone, Sumeet K Sodhi, Adrienne K Chan

**Affiliations:** ^1^ Dignitas International Zomba Malawi; ^2^ Dalla Lana School of Public Health University of Toronto Toronto Canada; ^3^ Zomba Central Hospital Malawi Ministry of Health Zomba Malawi; ^4^ Baylor College of Medicine Children's Foundation Malawi Lilongwe Malawi; ^5^ Department of Epidemiology Mailman School of Public Health Columbia University New York NY USA; ^6^ Department of Family and Community Medicine University Health Network University of Toronto Toronto Canada; ^7^ Division of Infectious Diseases Department of Medicine Sunnybrook Health Sciences Centre University of Toronto Toronto Canada

**Keywords:** HIV care continuum, adolescents, retention, differentiated care

## Abstract

**Introduction:**

There are numerous barriers to the care and support of adolescents living with HIV (ALHIV) that makes this population particularly vulnerable to attrition from care, poor adherence and virological failure. In 2010, a Teen Club was established in Zomba Central Hospital (ZCH), Malawi, a tertiary referral HIV clinic. Teen Club provides ALHIV on antiretroviral treatment (ART) with dedicated clinic time, sexual and reproductive health education, peer mentorship, ART refill and support for positive living and treatment adherence. The purpose of this study was to evaluate whether attending Teen Club improves retention in ART care.

**Methods:**

We conducted a nested case–control study with stratified selection, using programmatic data from 2004 to 2015. Cases (ALHIV not retained in care) and controls (ALHIV retained in care) were matched by ART initiation age group. Patient records were reviewed retrospectively and subjects were followed starting in March 2010, the month in which Teen Club was opened. Follow‐up ended at the time patients were no longer considered retained in care or on 31 December 2015. Cases and controls were drawn from a study population of 617 ALHIV. Of those, 302 (48.9%) participated in at least two Teen Club sessions. From the study population, 135 (non‐retained) cases and 405 (retained) controls were selected.

**Results:**

In multivariable analyses, Teen Club exposure, age at the time of selection and year of ART initiation were independently associated with attrition. ALHIV with no Teen Club exposure were less likely to be retained than those with Teen Club exposure (adjusted odds ratio (aOR) 0.27; 95% CI 0.16, 0.45) when adjusted for sex, ART initiation age, current age, reason for ART initiation and year of ART initiation. ALHIV in the age group 15 to 19 were more likely to have attrition from care than ALHIV in the age group 10 to 14 years of age (aOR 2.14; 95% CI 1.12, 4.11).

**Conclusions:**

This study contributes to the limited evidence evaluating the effectiveness of service delivery interventions to support ALHIV within healthcare settings. Prospective evaluation of the Teen Club package with higher methodological quality is required for programmes and governments in low‐ and middle‐income settings to prioritize interventions for ALHIV and determine their cost‐effectiveness.

## 1 Introduction

While significant progress has been made towards reduction of HIV infection and deaths worldwide, inequity has persisted in the headway made towards these goals 1. Contrary to the decreasing number of HIV‐related deaths among children under the age of ten and young adults aged 20 to 29, HIV‐related deaths among adolescents (aged 10 to 19) continue to rise and in Africa HIV is now the leading cause of death in this age group 1. Adolescents living with HIV (ALHIV) may have acquired HIV through sexual contact, or could have acquired it perinatally or during breastfeeding and survived into adolescence 2. The adolescent years are a critical time for intervention as ALHIV come to terms with their diagnosis and transition from child to adult physically, emotionally and psychologically 3.

Studies comparing treatment outcomes in adolescents and young adults to older adults have shown poorer outcomes in terms of virologic failure and retention in care 4. There is a multitude of social barriers to the care and support of ALHIV that makes this population particularly vulnerable to attrition from care, poor adherence and treatment failure. ALHIV in Malawi may be single or dual parent orphans, forcing them to meet the daily demands of living on their own 5. Antiretroviral treatment (ART) adherence may be compromised when an adolescent also has to be responsible for their health, food security, livelihood, and shelter. Stigma around HIV from peers at school or in their community may also compromise ART adherence, as ALHIV without peer support can feel isolated 6, 7. Disclosing HIV status may be difficult, creating a barrier to developing supportive friendships and peer relationships.

With the roll out of universal test and treat, increasing numbers of ALHIV will be starting ART and requiring adolescent‐appropriate ART services 8. There is little HIV‐specific or general adolescent evidence however to inform the most effective service delivery interventions and approaches for this population 9. In a review of twenty‐one research studies measuring HIV medication adherence (using subjective, pharmacologic and physiological indicators) and interventions to address adherence among ALHIV aged 13 to 24, overall rates of adherence were 28.3% to 69.8% before the interventions started 10. A systematic review which examined service delivery interventions aimed at improving retention in care and adherence to ART for ALHIV, concluded from a limited number of studies that peer counselling and support, as well as improved accessibility to clinics for ALHIV and availability of youth‐friendly services were promising strategies that warranted further investigation 11.

“Teen Club,” a targeted psychosocial support intervention, uses these strategies and others to address the barriers faced by ALHIV in achieving optimal treatment outcomes. In 2010, a Teen Club was established at Tisungane Clinic in Zomba Central Hospital (ZCH), Malawi, a tertiary referral HIV clinic of the Malawi Ministry of Health (MOH). The Teen Club package provides ALHIV on ART with dedicated weekend clinic time, sexual and reproductive health education, disclosure support, peer mentorship, support for treatment adherence, and a positive space for peer interaction through facilitated sports, arts and games. The purpose of this operational research study was to evaluate whether participation in Teen Club was associated with greater retention in care for ALHIV.

## 2 Methods

### 2.1 Study design

We conducted a nested case–control study with stratified selection using routine programmatic data from 2004 to 2015 for ALHIV patients.

### 2.2 Study setting

Study participants were ART patients followed as part of the *Zomba District Observational Cohort Study* (Z‐OCS), described in detail by Agarwal *et al*. 12 and also registered at ZCH, a tertiary urban referral hospital (catchment area 3.1 million) for the South East Health Zone in Zomba, Malawi. The Z‐OCS collects information from standard Malawi MOH ART monitoring tools either digitized from paper records or collected via electronic medical records (EMR) if available. Patients receive care at ZCH if it is their closest facility, if they choose to attend clinic there, or if they require complex specialized care from expert clinicians. Otherwise primary HIV care in Zomba District has been decentralized. 13 At ZCH, EMR records were implemented in 2010. Retrospective review of patient records was used and subjects were followed starting in March 2010, the month in which Teen Club was initiated at ZCH. Follow‐up ended at the time patients were no longer considered retained in care or on 31 December 2015.

#### 2.2.1 Description of the Teen Club intervention

The Tisungane Clinic Teen Club was established as a collaboration between the Malawi MOH, Dignitas International, and the Baylor College of Medicine Clinical Centre of Excellence Malawi, at a tertiary referral HIV clinic of the MOH. The Teen Club model was adapted from the Baylor College of Medicine International Pediatric AIDS Initiative – Center of Excellence Curriculum 13 and modified for a public sector HIV clinic setting run by MOH clinical staff in MOH clinic space, with partner support. Table 1. provides a summary of the differences between ALHIV who receive care in Teen Club versus standard of care. Teen Club provides ALHIV on ART with specialized and dedicated clinic time on Saturdays to eliminate school absenteeism. While a clinician provides specialized clinical care, a designated (non‐physician i.e. nurse, nursing aid, lay community health worker, expert client) staff member leads educational activities as well as crafts, sports and games. Clinicians have had training and mentorship in pediatric HIV care and nursing staff and lay health staff have been trained in the Teen Club Curriculum 13. Teens collect their medications from nurses and are assessed for adherence and provided individualized peer counselling and support as necessary. Teens that require additional clinical assessment (e.g. treatment for opportunistic infection, management of treatment failure) are referred for a clinician visit. Small group sessions are conducted (age stratified) for sexual and reproductive health education, as well as adherence support. Positive space and time occur concurrently to clinic visits while teens wait for their individualized clinic or adherence counselling visits through facilitated art and drama sessions and youth targeted games and sport. While not initially a part of programming (from 2010 to 2014), lunch is now provided at the end of each session. Teen club sessions are held monthly on Saturday mornings and attendees generally participate several hours in the joint activities. Adolescents are eligible for participation in Teen Club if they are between the ages of 10 and 19, are on ART, and have had their HIV status disclosed to them. ALHIV are referred to Teen Club by either an ART clinician or a nurse to ensure the disclosure process has been completed. The Teen Club is staffed by the regular ART clinicians and nurses who work on a rota and are accordingly compensated for overtime work in alignment with human resources policy at the MOH or at the non‐governmental organization (NGO).

**Table 1:** Description of standard of care for ALHIV compared with Teen Club programming for ALHIV registered at ZCH

	Standard of care 18	Teen Club
What?	ART prescribing ART refill by nurses Clinical review Family planning Labs	ART prescribing ART refill by nurses Clinical review Family planning Labs Structured adolescent‐focused group adherence counselling activities 13 Individualized psychosocial support 13 Activities promoting positive peer‐based social interactions: sports, games, dramas, dance, music, arts and crafts 13 Lunch (since 2014 only)
When?	Monday to Friday 8 AM to 5 PM every month for first 6 months then every 2 to 3 months pending adherence	Saturdays 8 AM to 1 PM every month or every 2 months pending adherence
Where?	Tisungane Clinic	Tisungane Clinic, ZCH open auditorium space near the clinic, and grounds for sports activities
Who?	5 Nurses (allowed to prescribe and refill ART) 5 Clinicians (clinical officers and physicians) for advance clinical review 2 ART counsellors and clerks 7 Expert Clients (to support adherence counselling ART clerks and act as peer navigators) *The same staff, staff both standard of care and Teen Club, except the ALHIV Expert Clients	3 Nurses (allowed to prescribe and refill ART) 2 Clinicians (clinical officers and physicians) for advance clinical review 1 ART counsellors and clerks 3 Expert Clients (to support adherence counselling ART clerks and act as peer navigators) 5 ALHIV Expert Clients (for peer to peer support; most often ALHIV transitioning to adult care)

ART, antiretroviral treatment; ZCH, Zomba Central Hospital; ALHIV, adolescents living with HIV.

### 2.3 Study participants

**Figure 1:**
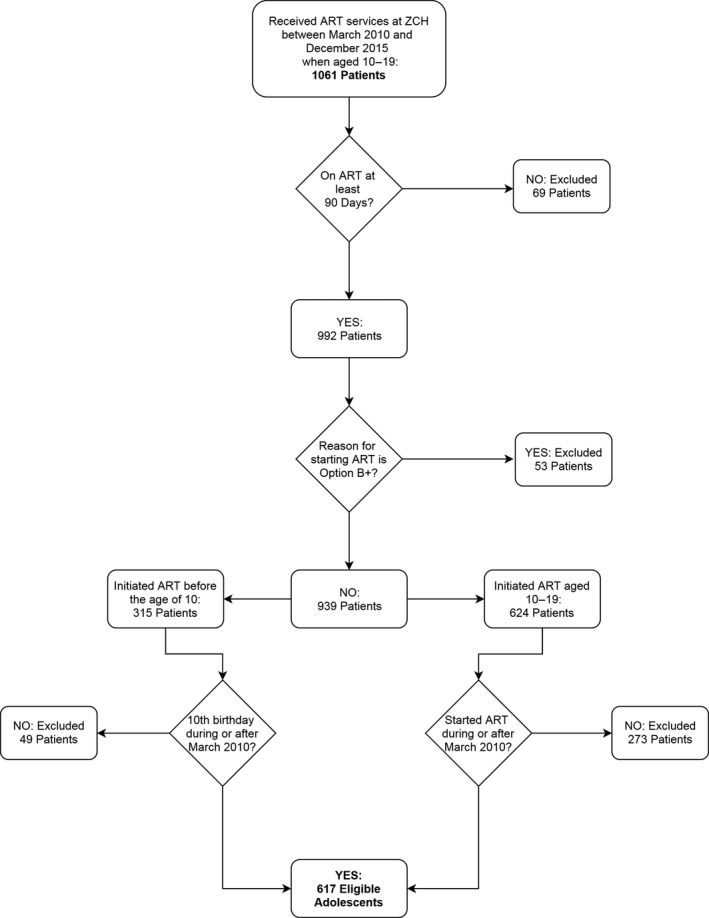
Study participants.

ALHIV were eligible for inclusion in the analysis if they initiated ART between the ages of 10 and 19 on or after March 2010 or if they initiated ART before the age of ten but turned ten during or after March 2010, and had a minimal follow‐up time of three months. Participants were excluded from the analysis if the reason for starting ART was pregnancy or breastfeeding as including these teens would skew the demographics of the study pool to older adolescent females. By excluding this population with different ART initiation criteria and clinical needs, we created a study pool most representative of the population that might attend Teen Club (see Figure 1).

Case and control selection was stratified by age at initiation of ART: under 10, 10 to 14, or 15 to 19 years of age. The total cohort of non‐retained ALHIV at time of data extraction was selected as cases. Controls were selected at a 3:1 ratio to cases by incidence density sampling, where observation start time was the date the subjects became eligible for teen club (date of ART registration for patients starting ART after the age of 10 or the 10th birthday for patients starting ART before the age of ten). Three controls was the maximum number that could be used per identified case. A greater than 3:1 ratio would leave cases with an insufficient number of matched participants to select controls from. Because incidence sampling was used, controls were selected from all adolescents who were retained in care at the same follow‐up time, regardless of whether or not they were future cases. This means that adolescents selected as controls at one point could subsequently be selected as cases at a later point. So, for every one case, three controls were selected from the same initiation age group at the same number of days after initiating ART and patients were assessed for exposure to Teen Club at that selection time.

### 2.4 Variables and data sources

All data in the Z‐OCS database was extracted as secondary data from routinely collected MOH patient monitoring and evaluation records 12.

#### 2.4.1 Sex

Patient's sex (male or female) as reported on ART master cards or in the EMR.

#### 2.4.2 ART initiation age

Age at time of starting ART for the first time: <10, 10 to 14, or 15 to 19 years of age.

#### 2.4.3 Current age

Age at time of entering in the study: 10 to 14, 15 to 19, or ≥20 years of age. Those ≥20 were 10 to 19 years of age and receiving ART at ZCH after the March 2010.

#### 2.4.4 ART reason

Primary reason for starting ART. This was deduced using a standard algorithm based on Malawi MOH guidelines for the year in which the ALHIV started ART. Clinical guidelines were published in 2003 14, 2006 15, 2008 16, 2011^17^, and 2014 18. While definitions and diagnostic criteria changed over the years, some consistency remained: all patients who presented with Stage III or IV classification of HIV based on World Health Organization (WHO) disease classification guidelines at that time would have their ART reason as WHO Stage III/IV, regardless of CD4 count. If the patient presented with WHO Stage I/II but had a CD4 count under the defined threshold at the time, the ART reason would be classified as CD4 count, (prior to 2011 and the introduction of Option B+, regardless of pregnancy/breastfeeding status). After 2011, when Option B+ was initiated in Malawi 17, patients would be given the ART reason of pregnancy or breastfeeding if she was pregnant or breastfeeding regardless of clinical or immunological status.

#### 2.4.5 Initiation year

The year in which the ALHIV started ART, using the categories 2004 to 2005, 2006 to 2007, 2008 to 2010, 2011 to 2013, 2014 to 2015, based on guidelines changes over time.

#### 2.4.6 Cumulative treatment outcome

The reported status (transferred out, alive on ART, lost to follow up (LTFU), stopped or died) at time of data extraction were defined as follows: a) “transferred out” – documented that patient has moved and transferred to another facility (otherwise classified as defaulted if transfer was not documented); b) “alive on ART” – patient is receiving ART and is not known to have been LTFU, died, or transferred to another facility; c) “LTFU” – patient did not return to the clinic within two months after (s)he was expected to have run out of ART, and is not known to have transferred out, stopped treatment, or died; the standardized MOH tools refer to these patients as “defaulted” d) “stopped” – patient is alive but is known to have stopped treatment for any reason; e) “died” – patient is known to have died for any reason, based on report by guardians, health facility staff, or from routine LTFU tracing by MOH staff.

#### 2.4.7 Outcome of interest

The outcome of interest for this study was non‐retention in ART care. Patients were considered “not retained in care” (cases) if their status at time of data extraction was “LTFU,” “stopped” or “died.”

#### 2.4.8 Teen Club (exposure)

Exposure to Teen Club was deduced based on visit dates of adolescents. All patient visits at ZCH after March 2010 were recorded in the EMR system. Patient‐level visit dates were extracted and compared to Teen Club session dates. Teen were considered to have attended, and thus been exposed to Teen Club if they had received ART services on at least two Teen Club Saturdays before selection as a case or control. All other (non‐Teen Club) ART clinic days are on Monday to Friday. Since adolescents often do not start attending Teen Club until days or years after initiating ART, we compared a patient's exposure to Teen Club sessions at the time of selection into the study as a case or control. Even if a participant attended Teen Club at some point during the follow‐up time, this participant could be considered unexposed at the time of selection as a control if selected at a follow‐up time prior to this participant's Teen Club sessions.

### 2.5 Statistical analysis

Descriptive statistics were performed to summarize the demographic and treatment characteristics of the study population. Chi‐square tests were used to compare characteristics between subgroups. The proportion of visits that were Teen Club versus non‐Teen Club visits for attenders after their first Teen Club session was calculated by dividing the total number of Teen Club visits by the total number of visits after and including the first Teen Club visit.

Bivariate logistic regression was used to assess the association between selected variables and non‐retention. Multivariable logistic regression was performed to measure the association between Teen Club exposure and non‐retention; adjusted for sex, age at the time of selection into the study, ART initiation age, reason for ART initiation and year of ART initiation. All statistical analysis was performed, using Stata 13 (STATA Corp., College Station, TX, USA).

### 2.6 Ethics

Ethics approval was obtained for the Z‐OCS study from the National Health Sciences Research Committee of Malawi and the University Health Network Research Ethics Board, University of Toronto, Canada. We did not obtain individual informed consent as we analysed fully anonymized routinely collected (secondary) program data.

## 3 Results

### 3.1 Study pool

The cases and controls were drawn from a total of 617 ALHIV as shown in Figure 1. Of those 302 (48.9%) participated in at least two Teen Club sessions. The median number of total Teen Club visits among these teens was 12 (data not shown). In this study pool, there were proportional differences in sex, year of ART initiation and cumulative treatment outcomes between those attending and not attending Teen Club (Table 2).

**Table 2:** Demographic and treatment characteristics of study pool

Variables	Non‐Teen Club	Teen Club^a^	Total	*p*‐value
	(n=315)	(n=302)	N=617	(X^2^)
	n (%)	n (%)		
Sex				
Male	122 (38.7)	144 (47.7)	266 (43.1)	
Female	193 (61.3)	158 (52.3)	351 (56.9)	0.03
ART initiation age				
<10	145 (46.0)	121 (40.1)	266 (43.1)	
10 to 14	103 (32.7)	105 (34.8)	208 (33.7)	
15 to 19	67 (21.3)	76 (25.2)	143 (23.2)	0.29
Current age^b^				
10 to 14	147 (46.7)	143 (47.4)	290 (47.0)	
15 to 19	120 (38.1)	124 (41.1)	244 (39.6)	
20 to 24	48 (15.2)	35 (11.6)	83 (13.5)	0.39
ART reason				
WHO Stage III/IV	204 (64.8)	188 (62.3)	392 (63.5)	
CD4 count	92 (29.2)	89 (29.5)	181 (29.3)	
Other/unknown/ missing	19 (6.0)	25 (8.3)	44 (7.1)	0.74
Initiation year				
2004 to 2005	2 (0.6)	3 (1.0)	5 (0.8)	
2006 to 2007	30 (9.5)	37 (12.3)	67 (10.9)	
2008 to 2010	151 (47.9)	103 (34.1)	254 (41.2)	
2011 to 2013	81 (25.7)	105 (34.8)	186 (30.2)	
2014 to 2015	51 (16.2)	54 (17.9)	105 (17.0)	0.01
Cumulative treatment outcome^c^				
Transferred out	48 (15.2)	45 (14.9)	93 (15.1)	
Retained (On ART)	156 (49.5)	233 (77.2)	389 (63.1)	<0.01
Defaulted	97 (30.8)	19 (6.3)	116 (18.8)	
Stopped	1 (0.3)	0 (0.0)	1 (0.2)	
Died	13 (4.1)	5 (1.7)	18 (2.9)	
Total not retained	111 (35.2)	24 (7.9)	135 (21.9)	<0.01

ART, antiretroviral treatment; WHO, World Health Organization.

a Subjects attended two or more Teen Club sessions.

b Current age=age as of 31 December 2015; Those ≥20 were in the age category 10 to 19 and receiving ART at ZCH after March 2010.

c Cumulative outcome=status as of the time of study with censoring date 31 December 2015.

Table 3 contains more detailed information about the attendance patterns for Teen club. Among Teen Club attenders, 79 (26.2%) started Teen Club within three months of starting ART, 52 (17.2%) had been on ART for 4 to 12 months, 31 (10.3%) between 13 and 24 months and 123 (40.7%) >24 months before starting Teen Club. There were 17 (5.6%) that had attended Teen Club before starting ART (in pre‐ART care). The majority, 197 (65.2%) started Teen Club between the ages of 10 and 14.

**Table 3:** Characteristics for Teen Club attenders in study pool

	n (%)
	(n=302)
Time on ART before starting Teen Club	
Started Teen Club before ART initiation	17 (5.6)
Within 1 month after ART initiation	30 (9.9)
2 to 3 months after ART initiation	49 (16.2)
4 to 12 months after ART initiation	52 (17.2)
13 to 24 months after ART initiation	31 (10.2)
24+ months after ART initiation	123 (40.7)
Age at *first* Teen Club visit	
<10^a^	36 (11.9)
10 to 14	197 (65.2)
15 to 19	67 (22.2)
20	2 (0.7)
Proportion of visits after first Teen Club visit that were Teen Club sessions: categories	
<75%	58 (19.2)
75% to 89%	91 (30.1)
90% to 99%	70 (23.2)
100%	83 (27.5)

ART, antiretroviral treatment.

a The majority of the teens who had their first Teen Club visit before the age of ten had it at age nine, and birthdates were often imprecise.

When exploring whether Teen Club members attended Teen Club or non‐Teen Club ART visits after their first Teen Club session, we found that 84.7% (SD 18.2) of subsequent visits were Teen Club ART visits (data not shown). The majority, 161 (57.6%), had 75% to 99% of their subsequent ART visits as Teen Club sessions. Only 20 (6.6%, data not shown) had less than 50% of their visits as Teen Club sessions, and 83 (27.5%) had all their subsequent visits as Teen Club visits.

### 3.2 Characteristics of cases and controls

From the study pool, 135 (non‐retained) cases and 405 (retained) controls were selected, matched on ART initiation age group. Demographic and treatment characteristics of cases and controls can be found in Table 4. There were no significant proportional differences in sex, ART initiation age, current age, reason for ART initiation or year of ART initiation between cases and controls. Teen Club exposure was higher among those retained versus those not retained (34.6% vs. 17.8%; *p*<0.01).

**Table 4:** Demographic and treatment characteristics of selected study participants by retention status

Variables (cases:control=3:1)	Retained (control n=405) n (%)	Not retained (cases n=135) n (%)	Total N=540	*p*‐value (χ^2^)
Sex				
Male	155 (38.3)	56 (41.5)	211 (39.1)	0.51
Female	250 (61.7)	79 (58.5)	329 (60.9)	
ART initiation age				
<10	123 (30.4)	41 (30.4)	164 (30.4)	1.0
10 to 14	165 (40.7)	55 (40.7)	220 (40.7)	
15 to 19	117 (28.9)	39 (28.9)	156 (28.9)	
Age at selection^a^				
10 to 14	217 (53.6)	68 (50.4)	285 (52.8)	0.80
15 to 19	164 (40.5)	58 (43.0)	222 (41.1)	
≥20	24 (5.9)	9 (6.7)	33 (6.1)	
ART reason				
WHO Stage III/IV	255 (63.0)	79 (58.5)	334 (61.9)	0.64
CD4 count	135 (33.3)	51 (37.8)	186 (34.4)	
Other/unknown	15 (3.7)	5 (3.7)	20 (3.7)	
Initiation year				
2004 to 2005	6 (1.5)	0	6 (1.1)	0.45
2006 to 2007	40 (9.9)	14 (10.4)	54 (10.0)	
2008 to 2010	187 (46.2)	63 (46.7)	250 (46.3)	
2011 to 2013	143 (35.3)	44 (32.6)	187 (34.6)	
2014 to 2015	29 (7.2)	14 (10.4)	43 (8.0)	
Attended 2 or more Teen Club sessions				
No	242 (59.8)	111 (82.2)	353 (65.4)	<0.01
Yes	163 (40.3)	24 (17.8)	187 (34.6)	

ART, antiretroviral treatment; WHO, World Health Organization.

a Age at selection=age at the time of selection as a case or a control; those >20 were in the age category 10 to 19 and receiving ART at ZCH after the year 2010.

**Table 5:** Bivariate and multivariable logistic regression of patient characteristics, Teen Club exposure and non‐retention

Variables	Crude OR (OR)^b^ [95% CI]	Adjusted^a^ OR (aOR)^b^ [95% CI]
Teen Club exposure		
No	1	1
Yes	0.32 [0.20, 0.52]	0.27 [0.16, 0.45]
Sex		
Male	1	1
Female	0.87 [0.59, 1.30]	0.80 [0.52, 1.23]
Current age		
10 to 14	1	1
15 to 19	1.13 [0.75, 1.69]	2.14 [1.12, 4.11]
≥20	1.20 [0.53, 2.70]	2.73 [0.86, 8.55]
ART initiation age		
<10	1	1
10 to 14	1 [0.63, 1.60]	0.76 [0.39, 1.48]
15 to 19	1 [0.60, 1.66]	0.47 [0.18, 1.24]
Reason for initiating ART		
WHO Stage III/IV	1	1
CD4 count	1.22 [0.81, 1.84]	1.47 [0.94, 2.33]
Other/unknown	1.08 [0.38, 3.05]	0.52 [0.14, 1.86]
Year of ART initiation		
2004 to 2005	‐	‐
2006 to 2007	1.04 [0.53, 2.03]	1.46 [0.66, 3.23]
2008 to 2010	1	1
2011 to 2013	0.91 [0.59, 1.42]	1.11 [0.67, 1.85]
2014 to 2015	1.43 [0.71, 2.88]	2.86 [1.14, 7.22]

ART, antiretroviral treatment; WHO, World Health Organization; aOR, adjusted odds ratio.

a Adjusted for all variables in the model.

b An odds ratio <1 indicates a lower odds of attrition from care.

### 3.3 Associations of patient characteristics and Teen Club exposure with non‐retention

In bivariate logistic regression analysis, only Teen Club exposure was associated with non‐retention (OR 0.32; 95% CI 0.20 to 0.52). In multivariable analyses, Teen Club exposure, age at the time of selection and year of ART initiation were independently associated with attrition. ALHIV with no Teen Club exposure were less likely to be retained than those with Teen Club exposure (adjusted odds ratio (aOR) 0.27; 95% CI 0.16, 0.45) when adjusted for sex, ART initiation age, current age, reason for ART initiation and year of ART initiation. ALHIV in the age group 15 to 19 were more likely to have attrition from care than ALHIV in the age group 10 to 14 years of age (aOR 2.14; 95% CI 1.12, 4.11) when adjusted for sex, ART initiation age, reason for ART initiation, year of ART initiation and Teen Club exposure.

## 4 Discussion

The results of this study provide promising evidence that the “Teen Club,” an adolescent‐centred psychosocial support intervention is effective in reducing attrition from care in ALHIV. We showed that exposure to the Teen Club package is associated with a 3.7‐times lower odds of attrition than not being exposed to Teen Club. This study contributes to the limited existing evidence 9 evaluating youth‐friendly services as promising strategies in improving retention in care for ALHIV.

In line with other literature 19, 20, 21, 22, 23, 24, 25, this study further reinforces that older ALHIV (15 to 19 years) have a higher risk (two‐times higher) of attrition from care than younger ALHIV (10 to 14 years) and highlights the need for age‐group specific programing and transitioning protocols that start in older adolescence. A recent situational analysis of 218 facilities in 23 sub‐Saharan African countries showed that two‐thirds attended to ALHIV with adults and children, and 50% of clinics had no transitioning protocols for older adolescents, despite the fact that non‐adherence was reported as a key challenge 25.

We chose to undertake a nested case–control study to attempt to address the issues of retention bias by accounting for time‐dependent exposure and also to take into account time on ART prior to Teen Club. There are obvious limitations for a retrospective operational research study due to the inability to capture some important information if it was not available in the standardized monitoring and evaluation forms of the MOH e.g. perinatal versus horizontal (i.e. behavioural) transmission, distance from clinic.

The lack of Teen Club registration or attendance records also provided challenges in defining the exposure. We assumed attendance based on clinic visit documentation on Teen Club days (Saturdays), however, there were also Saturday visits by some adolescents on non‐Teen Club Saturdays and therefore we could not assume that any Saturday visit was a Teen Club visits which was why two visits were chosen as the exposure threshold. There may be some selection bias in Teens who have chosen to attend Teen Club for retention and treatment self‐efficacy that we cannot account for with the data available.

There remains a lack of evidence for the effectiveness of service delivery interventions to support ALHIV within healthcare settings with the most comprehensive systematic review 11 noting that available evidence comes from a small number of studies with low to moderate methodological quality. Youth friendly models of care focusing on supporting adolescent and youth self‐efficacy on ART have been implemented, and in some cases at scale. Our program has been adapted from the Baylor Teen Club model 13, 26 which has been implemented at scale in Malawi, and in countries supported by the Baylor Pediatric AIDS Initiative. Médecins sans Frontières 27, 28, 29 has also piloted a Youth Clubs program in Cape Town, including a structured interactive activity‐based session rooted in peer support that has been endorsed in the South African national adherence policy. Few interventions, however, have been described in the peer‐reviewed literature and evaluations have not looked at retention in care as an outcome 27, 28, 29, 30, 31, 32, 33, 34. This is one of the first studies to date to look at the impact of an adolescent focused differentiated model of care on ART retention.

Further qualitative research in particular around the barriers associated with retention in care, non‐adherence, the effect on Teen Club programming on these barriers, and disentangling the individual factors that may prompt ALHIV to choose standard of care versus Teen Club, would be valuable in evidence‐informed program modification. A recent cross sectional study 34 of a representative sample of ALHIV in the ZCH clinic and another large tertiary referral clinic in Malawi, looked at barriers and associated factors to self‐reported non‐adherence and found the most commonly reported barriers to adherence included forgetting (>90%), travel from home (14%), and “busy doing other things” (11%). Drinking alcohol in the past month, witnessing or experiencing violence in the home in the past year, and poor treatment self‐efficacy were each found to be independently associated with missing ART in the past week.

The absence of evidence and operations research around ALHIV targeted services, limits the ability of programmes and governments in high prevalence settings to prioritize interventions and determine their cost‐effectiveness. It also prevents monitoring impact of interventions on the health system, especially because resource intensive approaches applied at the individual patient level are unlikely to be generalizable in low‐income countries with health worker shortages. While this study demonstrates promising evidence supporting the efficacy of the Teen Club package, more rigorous prospective evaluation involving pragmatic trial design would be helpful to inform scalability.

## 5 Conclusions

Teen Club, a specialized psychosocial support intervention providing dedicated clinic time, health education and peer support and mentorship for ALHIV is associated with 3.7 times greater odds of retention compared with ALHIV who are not exposed to the intervention. More health services research into the management of ALHIV transitioning into adult care is urgently needed in sub‐Saharan Africa, with particular attention to differentiated models of care.

## Competing interests

The authors have no competing interests to declare.

## Authors’ Contributions

AKC, MvL, RM, MA and DG contributed to the concept and design of the study. AKC, CG, JN, VS, ET, TB, AM, SH and DG contributed to the implementation of the intervention. CG, JN, VS, VB, ET, TB and SKS contributed to acquisition of data. RM and MvL contributed to data analysis and/or interpretation of the data. RM, MvL and AKC drafted the first manuscript. CG, JN,VS, VB, ET, TB, MA, AM, SH, DG and SKS reviewed and suggested revisions. All authors reviewed and approved the final manuscript.

## Funding

The Zomba Central Hospital Tisungane Clinic Teen Club program from 2010 to 2015 was funded by Dignitas International, the Malawi National AIDS Commission, the United States Agency for International Development (4‐612‐17‐117‐R). The Zomba District Observational Cohort Study was funded by the Canadian Institutes for Health Research (HHP‐111405 and HIB‐126784) from 2011 to 2015.

## References

[1] UNAIDS . The Joint United Nations Programme on HIV/AIDS. All In to #EndAdolescentAIDS. 2015 Available from: http://www.unaids.org/sites/default/files/media_asset/20150217_ALL_IN_brochure.pdf

[2] Idele P , Gillespie A , Porth T , Suzuki C , Mahy M , Kasedde S , et al. Epidemiology of HIV and AIDS among adolescents: current status, inequities, and data gaps. J Acquir Immune Defic Syndr. 2014;66:S144–53. 24918590 10.1097/QAI.0000000000000176

[3] Lowenthal ED , Bakeera‐Kitaka S , Marukutira T , Chapman J , Goldrath K , Ferrand RA . Perinatally acquired HIV infection in adolescents from sub‐Saharan Africa: a review of emerging challenges. Lancet Infect Dis. 2014;14:627–39. 24406145 10.1016/S1473-3099(13)70363-3 PMC4074242

[4] Evans D , Menezes C , Mahomed K , Macdonald P , Untiedt S , Levin L , et al. Treatment outcomes of HIV‐infected adolescents attending public‐sector HIV clinics across Gauteng and Mpumalanga, South Africa. AIDS Res Hum Retroviruses. 2013;29:892–900. 23373540 10.1089/aid.2012.0215 PMC3653371

[5] Hosegood V , Floyd S , Marston M , Hill C , McGrath N , Isingo R , et al. The effects of high HIV prevalence on orphanhood and living arrangements of children in Malawi, Tanzania, and South Africa. Popul Stud (Camb). 2007;61:327–36. 17979006 10.1080/00324720701524292 PMC2216069

[6] Mutwa PR , Van Nuil JI , Asiimwe‐Kateera B , Kestelyn E , Vyankandondera J , Pool R , et al. Living situation affects adherence to combination antiretroviral therapy in HIV‐infected adolescents in Rwanda: a qualitative study. PLoS ONE. 2013;8(4):e60073. 23573232 10.1371/journal.pone.0060073 PMC3616046

[7] Kim MH , Mazenga AC , Yu X , Devandra A , Nguyen C , Ahmed S , et al. Factors associated with depression among adolescents living with HIV in Malawi. BMC Psychiatry. 2015;15:264. 26503291 10.1186/s12888-015-0649-9 PMC4624356

[8] World Health Organization . Consolidated strategic information guidelines for HIV in the health sector. Geneva. Switzerland: World Health Organization; 2015 [cited 2017 Jun 5]. Available from: http://who.int/hiv/pub/guidelines/strategic-information-guidelines/en/ 26110192

[9] Judd A , Sohn AH , Collins IJ . Interventions to improve treatment, retention and survival outcomes for adolescents with perinatal HIV‐1 transitioning to adult care: moving on up. Curr Opin HIV AIDS. 2016;11:477–86. 27272537 10.1097/COH.0000000000000302

[10] Reisner SL , Mimiaga MJ , Skeer M , Perkovich B , Johnson CV , Safren SA . A review of HIV antiretroviral adherence and intervention studies among HIV‐infected youth. Top HIV Med. 2009;17:14–25. 19270345 PMC3752381

[11] MacPherson P , Munthali C , Ferguson J , Armstrong A , Kranzer K , Ferrand RA , et al. Service delivery interventions to improve adolescents’ linkage, retention and adherence to antiretroviral therapy and HIV care. Trop Med Int Health. 2015;20:1015–32. 25877007 10.1111/tmi.12517 PMC4579546

[12] Agarwal M , Bourgeois J , Sodhi S , Matengeni A , Bezanson K , van Schoor V , et al. Updating a patient‐level ART database covering remote health facilities in Zomba district, Malawi: lessons learned. Public Health Action. 2013;3(2):175–9. 26393023 10.5588/pha.12.0096 PMC4463119

[13] Baylor International Pediatric AIDS Initiative . Baylor College of Medicine International Pediatric Pediatric AIDS Initiative – Malawi Teen Club Curriculum. A Resource for Groups working with Adolescents Living with HIV. February 2012. Lilongwe, Malawi.

[14] Malawi Ministry of Health . Treatment of AIDS: Guidelines for the use of antiretroviral therapy in Malawi. 2003 Available from: https://searchworks.stanford.edu/view/6715377

[15] Malawi Ministry of Health . Treatment of AIDS: Guidelines for the use of antiretroviral therapy in Malawi. 2006 Available from: https://searchworks.stanford.edu/view/6688644

[16] Malawi Ministry of Health . Treatment of AIDS: Guidelines for the use of antiretroviral therapy in Malawi. 2008 Available from: http://apps.who.int/medicinedocs/documents/s18803en/s18803en.pdf

[17] Malawi Ministry of Health . Clinical Management of HIV in Children and Adults. 2011 Available from: http://apps.who.int/medicinedocs/documents/s18802en/s18802en.pdf

[18] Malawi Ministry of Health . Clinical Management of HIV in Children and Adults. 2014 Available from: http://www.emtct-iatt.org/wp-content/uploads/2015/09/Malawi-HIV-Guidelines-2014.pdf

[19] Auld F , Agolory SG , Shiraishi RW , Wabwire‐Mangen F , Kwesigabo G , Mulenga M , et al. Antiretroviral therapy enrollment characteristics and outcomes among HIV‐infected adolescents and young adults compared with older adults – Seven African countries, 2004‐2013. Washington, DC: Centers for Disease Control and Prevention – Morbidity and Mortality Weekly Report 2014. PMC5779521 25426651

[20] Lamb MR , Fayorsey R , Nuwagaba‐Biribonwoha H , Viola V , Mutabazi V , Alwar T , et al. High attrition before and after ART initiation among youth (15‐24 years of age) enrolled in HIV care. AIDS. 2014;28:559–68. 24076661 10.1097/QAD.0000000000000054 PMC4517438

[21] Koech E , Teasdale CA , Wang C , Fayorsey R , Alwar T , Mukui IN , et al. Characteristics and outcomes of HIV‐infected youth ad young adolescents enrolled in HIV care in Kenya. AIDS. 2014;28:2729–38. 25493599 10.1097/QAD.0000000000000473 PMC5098333

[22] Bygrave H , Mtangirwa J , Ncube K . Antiretroviral therapy outcomes among adolescents and youth in rural Zimbabwe. PLoS ONE. 2012;7:e52856. 23285204 10.1371/journal.pone.0052856 PMC3527625

[23] Ugwu R , Eneh A . Factors influencing adherence to paediatric antiretroviral therapy in Portharcourt, South‐South Nigeria. Pan Afr Med J. 2013;16:30. 24570791 10.11604/pamj.2013.16.30.1877 PMC3932123

[24] Dachew BA , Tesfahunegn TB , Birhanu AM . Adherence to highly active antiretroviral therapy and associate factors among children at the University of Gondar Hospital and Gondar Poly Clinic, Northwest Ethiopia: a cross‐sectional institutional based study. BMC Public Health. 2014;14:875. 25155293 10.1186/1471-2458-14-875 PMC4158084

[25] Lall P , Lim SH , Khairuddin N , Kamarulzaman A . Review: an urgent need to research on factors impacting adherence to and retention in care among HIV‐positive youth and adolescents from key populations. J Int AIDS Soc. 2015;18 Suppl 1:19393. 25724503 10.7448/IAS.18.2.19393 PMC4344535

[26] International AIDS Society . Differentiated Care: Facility‐based individual models‐Teen Clubs. Available from: http://www.differentiatedcare.org/Models/TeenClubs

[27] International AIDS Society . Differentiated Care: Health care worker managed groups‐Youth clubs. Available from: http://www.differentiatedcare.org/Models/YouthClubs

[28] Henwood R , Patten G , Barnett W , Hwang B , Metcalf C , Hacking D , et al. Acceptability and use of a virtual support group for HIV‐positive youth in Khayelitsha Cape Town, using the MXit social networking platform. AIDS Care. 2016;28(898):903. 10.1080/09540121.2016.1173638 27098208

[29] Wilkinson L , Moyo F , Henwood R , Runeyi P , Patel S , de Azevedo V , et al. Youth ART adherence clubs: Outcomes from an innovative model for HIV positive youth in Khayelitsha, South Africa. 21st International AIDS Conference, Durban, 18‐22 July 2016.

[30] McKenney A . Transferring the necessary economic, psychosocial, and self‐care skills needed for young adults living with HIV in Malawi. In: 2nd Adoelscent Transition Workshop, editor. 2016 Apr 6; Budapest, Hungary: Baylor College of Medicine; 2016.

[31] Nyabigambo A , Mulira JK , Atuyambe L , Babikako HM , Kambugu A , Ndoleriire C . Determinants of utilization of a no‐cost HIV transition clinic: a cross sectional study of young adults living with HIV/AIDS. Adolesc Health Med Ther. 2014;5:89–99. 24966709 10.2147/AHMT.S57950 PMC4043429

[32] Nyabigambo A , Mulira JK , Kambugu A . Transitional clinic utilization and general well‐being of Ugandan young adults living with HIV/AIDS. Value Health. 2014;17:A666. 10.1016/j.jval.2014.08.2454 27202437

[33] Patten GE , Wilkinson L , Conradie K , Isaakidis P , Harries AD , Edginton ME , et al. Impact on ART initiation of point‐of‐care CD4 testing at HIV diagnosis among HIV positive youth in Khayelitsha, South Africa. J Int AIDS Soc. 2013;16:18518. 23830642 10.7448/IAS.16.1.18518 PMC3702919

[34] Kim MH , Mazenga AC , Yu X , Ahmed S , Paul ME , Kazembe PN , et al. High self‐reported non‐adherence to antiretroviral therapy amongst adolescents living with HIV in Malawi: barriers and associated factors. J Int AIDS Soc. 2017;10:21437. 10.7448/IAS.20.1.21437 PMC5515061 28406275

